# Examination of Candidates for Assistant Surgeon

**Published:** 1913-05

**Authors:** 


EXAMINATION OF CANDIDATES FOR ASSISTANT SURGEON.—Treasury Department, United States Public Health Service, Washington, April 14, 1913.—Boards of commissioned medical officers will be convened to meet at the Bureau of Public Health Service, 3 B Street, SE., Washington, D. C., and at the Marine Hospitals at Boston, Mass., Chicago, Ill., New Orleans, La., and San Francisco, Cal., on Monday, May 5, 1913, and Monday, June 9, 1913, at 10 o'clock a. m., for the purpose of examining candidates for admission to the grade of assistant surgeon in the Public Health Service.

Candidates must be between 23 and 32 years of age, graduates of a reputable medical college, and must furnish testimonials from two responsible persons as to their professional and moral character. Service as internes in hospital for the insane or experience in the detection of mental diseases will be considered and credit given in the examination. Candidates must have had one year's hospital experience or two years' professional work.

Candidates must be not less than 5 feet, 4 inches, nor more than 6 feet, 2 inches, in height.

The examination usually covers a period of about 10 days.

For further information, or for invitation to appear before the board of examiners, address "Surgeon General, Public Service, Washington, D. C."

